# Plasma microRNA expression levels in HIV-1-positive patients receiving antiretroviral therapy

**DOI:** 10.1042/BSR20194433

**Published:** 2020-05-14

**Authors:** Jazmin Marquez-Pedroza, Jhonathan Cárdenas-Bedoya, María Cristina Morán-Moguel, Martha Escoto-Delgadillo, Blanca Miriam Torres-Mendoza, Alma Minerva Pérez-Ríos, Gracia Viviana González-Enriquez, Eduardo Vázquez-Valls

**Affiliations:** 1 Laboratorio de Inmunodeficiencias y Retrovirus Humanos, Centro de Investigación Biomédica de Occidente, Instituto Mexicano del Seguro Social, Guadalajara 44340, Jalisco, Mexico; 2 Doctorado en Farmacología, Centro Universitario de Ciencias de la Salud, Universidad de Guadalajara, Guadalajara 44350, Jalisco, Mexico; 3 Doctorado en Genética Humana, Centro Universitario de Ciencias de la Salud, Universidad de Guadalajara, Guadalajara 44340, Jalisco, Mexico; 4 Centro Universitario de Ciencias Biológicas y Agropecuarias, Universidad de Guadalajara, Zapopan 45200, Jalisco, Mexico; 5 Departamento de Disciplinas Filosófico, Metodológicas e Instrumentales, Centro Universitario de Ciencias de la Salud, Universidad de Guadalajara, Guadalajara 44340, Jalisco, Mexico; 6 Hospital Regional de Zona 110, Instituto Mexicano del Seguro Social, Guadalajara 44716, Jalisco, Mexico; 7 Director de Generación de Recursos Profesionales, Investigación y Desarrollo, Secretaria de Salud, Jalisco, Guadalajara 44100, Jalisco, Mexico

**Keywords:** drug resistance, HAART, HIV-1, miR-16-5p, miR-26a-5p, therapy failure

## Abstract

MicroRNAs (miRNAs/miRs) may serve as therapeutic agents or targets in diseases in which the expression of proteins plays an important role. The aim of the present study was to compare the expression levels of specific miRNAs, as well as their correlation with markers of response to antiretroviral (ARV) therapy, in patients with human immunodeficiency virus type 1 (HIV-1) infection with and without resistance to highly active antiretroviral therapy (HAART). Methods: miRNA assays were performed on plasma samples obtained from 20 HIV-1-positive patients. A total of ten patients were divided into two groups: HAART-responsive and HAART-resistant (*n*=5 per group). Commercial arrays were subsequently used to identify 84 miRNAs. A total of three differentially expressed miRNAs were selected and analyzed by quantitative PCR (qPCR). Five other patients were subsequently added to each group for a new relative expression analysis. The absolute expression level of the two miRNAs was obtained and compared using the Student’s *t* test. Receiver operating characteristic (ROC) curves were used to identify patients with antiretroviral therapy (ART) resistance.

**Results:** The array analysis revealed that miR-15b-5p, miR-16-5p, miR-20a-5p, miR-26a-5p, miR-126-3p and miR-150-5p were down-regulated in patients with HAART-resistance comparing with HAART-responsive. The expression levels of miR-16-5p, miR-26a-5p and miR-150-5p were confirmed using qPCR. The area under the ROC curve was 1.0 for the three miRNAs.

**Conclusions:** The lower expression levels of miR-16-5p and miR-26a-5p in patients with HAART-resistance suggested that these may serve as potential biomarkers for the identification of HAART-responsive patients.

## Introduction

The human immunodeficiency virus type 1 (HIV-1) is a retrovirus that causes the acquired immunodeficiency syndrome (AIDS). Approximately 74.9 million people have been infected with HIV since the beginning of the epidemic, of which ∼32 million have died from AIDS-related diseases. Until December 2018, ∼37.9 million people lived with HIV worldwide and only 23.3 million had access to antiretroviral therapy (ART) [1]. The number of antiretroviral (ARV) available for treatment of HIV infection has increased considerably in recent years; with the onset of highly active ART (HAART) in 1996, the life expectancy of HIV-positive people increased considerably [2,3]. However, the effectiveness of an ARV treatment scheme against virus replication depends on the effectiveness of each ARV and the number of genetic variations of HIV-1 that are required for the expression of virus resistance to ARV [4–6]. The increase in treatment failure due to the resistance of the virus to ARVs has been decreasing the number of highly active ARVs available to control the disease [6]. This decrease in effectiveness of ARVs has led to the search for new therapies that help control virus replication and disease progression, an alternative potential is the use of miRNAs [7].

MicroRNAs (miRNAs/miRs) are small non-coding RNAs of 20–25 nucleotides that modulate gene expression by transcript degradation and translational suppression [8]. miRNAs may serve as therapeutic agents or targets in diseases where the expression of specific proteins serves an important role in disease progression as cancer or viral infections. It has been reported that miRNAs may decrease the replication of the HIV-1 by silencing host-dependent factors and viral proteins involved in the progress of the disease [9–11]. Furthermore, miRNAs may influence in which cell types latency and active infection take place [9]. However, expression analyses revealed that the total number of miRNAs identified in each group of patients differs (ART-naïve AIDS patients, those on ART, and those developing drug resistance and failing ART) [11–13]. Additionally, miRNAs may be implicated in the resistance to ART [8], however, this has been poorly assessed [11,12].

HIV has been shown to develop drug resistance to ART [14]. While a number of previously published studies suggested that miRNAs affect HIV replication, few results have been corroborated [11,15,16]. The most consistent results revealed that HIV affects immune system-associated miRNAs, including miR-155, miR-150, miR-125b and miR-146a, which may serve as nonspecific biomarkers of immune responses [8,11,17]. Munshi et al. [11] evaluated miRNA profiles in plasma obtained from HIV-positive patients and revealed that miR-150 was down-regulated in those developing drug resistance and failing ART versus on ART. However, the roles of other miRNAs such as miR-16 have not been investigated in HIV. Establishing the roles of miRNAs in patients with ART resistance may aid the development of therapeutic and diagnostic strategies [18]. Therefore, the aim of the present study was to compare miRNA expression levels, as well as their correlations with markers of response to therapy, in HIV-1-positive patients with and without HAART resistance.

## Materials and methods

### Ethical approval

The present cross-sectional study was approved by the Research and Ethics Committee of Centro de Investigación Biomédica de Occidente (CIBO), Instituto Mexicano del Seguro Social (IMSS; approval no. R-2014-1305-11). Written informed consent was obtained from all participants. The presentstudy was conducted according to the seventh revision of the Declaration of Helsinki (2013).

### Patients and samples

A total of 20 male patients, between 20 and 50 years, HIV-1 positive, from Western Mexico who were admitted to the Laboratorio de Inmunodeficiencias y Retrovirus Humanos at CIBO-IMSS for the determination of ARV resistance genotype between January 2012 and December 2015 were included in the present study.

**Figure 1:**
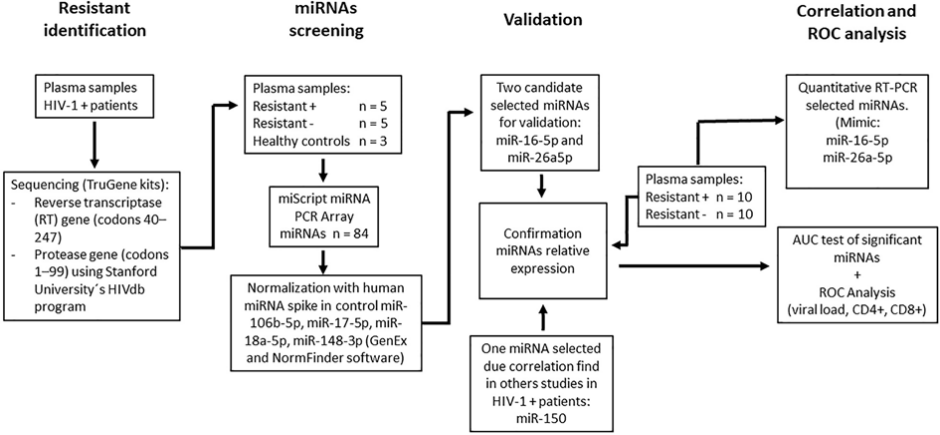
Process diagram The number of samples and each of the stages (resistant identification, miRNAs screening, validation and correlation and ROC analysis) of their processing are presented.

Clinical and demographic data of the patients were collected. The ART compliance questionnaire applicated was Morisky-Green et al. and validated in their Spanish version by Val-Jiménez et al. [19,20]. The sample size was calculated according to Kok et al., considering a power (Z_β_) = 0.8; effect size = 1.7 [21]. A total of ten patients participated in the first stage of the study, which involved relative expression analysis of miRNAs. The initial results were confirmed by analyzing the samples obtained from the remaining ten patients. The samples and processes used in the present study are presented in (Figure 1).

The inclusion criteria were as follows: (i) male patients > 18 years old; (ii) patients receiving HAART; (iii) patients with <12000 platelets/µl; (iv) patients with evidence of disease progression [>1000 HIV-1 RNA copies/ml or <500 cluster of differentiation (CD) 4^+^ T cells/µl]. The exclusion criteria included patients with concomitant hepatitis B or C, influenza, tuberculosis, diabetes, cancer or cardiovascular disease.

### Preparation of plasma samples

Peripheral venous blood was drawn from the median cubital vein of each patient using a 22-gauge 1.5-inch needle into two 7 ml EDTA-coated tubes [22]. The venipuncture was performed according to the World Health Organization guidelines on drawing blood from adults [23]. Plasma was obtained from the blood immediately after sample collection by two consecutive centrifugation steps. Blood was centrifuged at 200×***g*** for 15 min at 15°C. The supernatant was subsequently decanted and centrifuged at 1000×***g*** for 15 min at 15°C. The supernatant was transferred into cryotubes and stored at −80°C until further use. Samples that appeared hemolyzed with reddish coloration were discarded as recommended [24].

### Quantification of viral load, CD4^+^ and CD8^+^ cells

CD4^+^ and CD8^+^ cells were quantified by flow cytometry (Cytomics FC500; Beckman Coulter, Inc.) with CXP Software (version 2.3; Beckman Coulter, Inc.). The viral load was determined in plasma using Arthus® HI Virus-1 QS-RGQ kit (Qiagen Inc.), QIAsymphony® SP/AS sample extraction (Qiagen Inc.) and preparation apparatus and Rotor-Gene Q® real-time PCR machine (Qiagen Inc.). Conditions: reverse transcription of the RNA was done for 30 min at 50°C; initial activation of the hot-start enzyme was for 15 min at 95°C; amplification of the cDNA (denaturation: 95°C for 30s, annealing: 50°C for 60s and elongation; 62°C for 30s) for 50 cycles. Genotyping and miRNA analysis were performed for 1–2 days and 12–36 months following plasma extraction, respectively.

### Determination of ARV resistance genotype

Viral RNA for the genotyping and identification of mutations that confer resistance to ARVs was obtained from plasma samples using QIAamp viral RNA kits (Qiagen, Inc.) following the manufacturer’s instructions. RNA concentration was determined using a NanoDrop 2000/2000c spectrophotometer (Thermo Fisher Scientific, Inc.) and the ratio of absorbance at 260 and 280 nm was used to assess RNA purity. The RNA integrity was evaluated by electrophoresis on 1.5% agarose-formaldehyde gels.

The product of viral RNA extraction was used for sequencing the reverse transcriptase (RT) gene (codons 40–247) and protease gene (codons 1–99) using TruGene kits (Siemens AG). Direct sequencing of double-stranded DNA was performed on an automated sequencer. ARV therapy resistance was determined using Stanford University’s HIVdb program (hivdb.stanford.edu). After genotyping, ten plasma samples (five with HAART resistance and five without) were selected for the relative expression analysis of 84 miRNAs that were most relevant to pathophysiological conditions and were detectable and differentially expressed in serum, plasma, and other bodily fluids. After the first relative expression analysis, the groups with and without resistance to HAART were completed at ten samples each for the verification of the relative expression analysis and the quantification of the absolute expression.

### RNA isolation and cDNA synthesis

Total RNA was extracted from plasma samples using the miRNeasy Serum/Plasma kit (Qiagen, Inc.) for miRNA assays, following the manufacturer’s instructions. The concentration, purity and integrity of the RNA were assessed as aforementioned. Subsequently, all RNA samples were used for cDNA synthesis using the miScript ІІ RT kit (Qiagen, Inc.) following the manufacturer’s instructions, as follows: 4 μl of 5× miScript HiSpec Buffer (relative expression) or 5× miScript HiFlex Buffer (absolute expression), 2 μl of 10× Nucleics Mix, 10 μl RNase-free water, 2 μl miScript Reverse Transcriptase Mix, 2 μl Template RNA. Total reaction volumes were 20 μl each. The RT-PCR conditions were: one cycle for 60 min at 37°C to retrotranscription and 5 min at 95°C to inactivate miScript Reverse Transcriptase Mix.

### MiRNA arrays for relative expression

Screening of the 84 miRNAs was performed using the miScript miRNA PCR array (Qiagen, Inc.) and cDNA from RNA extracted from plasma sources, and a Rotor-Gene Q® PCR machine (Qiagen, Inc.). miScript primer assay (Qiagen, Inc.) and miScript SYBR Green PCR kits (Qiagen, Inc.) were used according to the manufacturer’s instructions. Briefly, 1100 μl of 2× QuantiTect SYBR Green PCR Master Mix were added, 220 μl of 10× miScript Universal Primer, 100 μl of template cDNA and 780 μl of RNase-free water at room temperature. The reaction volumes were 20 μl per well for a 100-well Rotor-Disc. The cycling conditions for real-time quantitative PCR (qPCR) were PCR initial activation for 15 min at 95°C, denaturation for 15 s at 94°C, annealing of 30 s at 55°C, an extension of 30 s at 70°C, for 40 cycles. The maximum number of quantification cycles (*C*_q_) was 32 as recommended [25,26]. The efficiency of miRNA isolation was monitored by the amount of spiked-in *Caenorhabditis elegans* miR-39 recovered according to the manufacturer’s instructions. Briefly, 3.5 μl miRNeasy Serum/Plasma Spike-in control (Qiagen, Inc.) (1.6 × 10^8^ copies/μl *C. elegans* miR-39) were added to 100 μl plasma. The efficiency of reverse transcription-qPCR (RT-qPCR) was assessed by measuring synthetic miRNA using positive PCR controls. All experiments were performed in duplicate and a no template control for RT-qPCR was included in each reaction batch.

### Normalization criteria

Due to the low expression levels of small nucleolar RNA and small nuclear RNA PCR controls in plasma, alternative strategies based on the algorithms of geNorm-implemented GenEx software (version 6; www.biomcc.com/genex-software.html) [27] and NormFinder (version 0.953; moma.dk/normfindersoftware), were used. Both programs evaluate the relative expression of data normalization by means of multiple internal control genes [28]. Due to the small sample size, the study was adhered to previous statistical normalization criteria and the following conditions: maintain homogeneous conditions by using the same standardized protocol for all samples, reduce freeze/thaw cycles, reduce batch effects by not mixing different experimental batches, add a exogenous synthetic RNA for technical normalization (exogenous controls), selection of endogenous controls through algorithms for the identification and selection of stable miRNAs candidates, data quality control, validation [29].

### MiRNAs for absolute expression (validation)

The plasma copy numbers of more expressed miRNAs in ten samples from each group were determined using qPCR. A standard curve was prepared for each miRNA using the miScript mimics serial dilution method (Qiagen). A total of 10^3^, 10^5^, 10^7^ and 10^9^ copies were used for each RT-qPCR, followed by qPCRs with the miScript Primer assay (Qiagen, Inc.).

### Data analysis

Data analysis for the relative expression levels of miRNAs were performed according to the manufacturer’s instructions using web-based software package (pcrdataanalysis.sabiosciences.com/mirna/arrayanalysis.php), the *C*_q_ comparative method was used and changes in the expression levels of miRNAs were calculated using the 2^–ΔΔ*C*_q_^ equation [30,31]. The expression data are presented as the mean ± the standard error of the mean. Correlation analysis was performed using the two-tailed Pearson correlation test. The sensitivity, specificity and area under the curve (AUC) were analyzed using SPSS software (version 21; IBM Corp.). Following the aforementioned normalization procedure, the Student’s *t* test was used to compare the mean expression levels of miRNAs between the two groups and the χ^2^ test was used to compare frequencies. *P*<0.05 was considered to indicate a statistically significant significance.

## Results

### Patient characteristics

All patients were aged between 20 and 50 years. No differences in patient characteristics, including age, viral load, CD4^+^ and CD8^+^ cell counts, duration of infection and HAART regimen used, were observed between the two groups.

Patient data, including immunological and clinical information, time elapsed since diagnosis, treatment time with HAART and treatment time with the last HAART scheme, are presented in (Table 1). The clinical and immunological criteria of the Center for Disease Control and Prevention were used to classify patients into stages [32]. In the group of patients with HAART resistance, seven were in stage 3 (acquired immune deficiency syndrome), two in stage 2 and one in stage 1. In the group of non-resistant patients, five were in stage 3, four in stage 2 and one in stage 1.

**Table 1:** General data for patients with non-resistance and resistance to HAART

	Resistance		Non-resistance	
	Mean	SD	Mean	SD
Age (yr)	36.8	16.7	41.5	4.9
CD4^+^ (cells/dl)	228.1	165.4	355.4	357.6
CD8^+^ (cells/dl)	718.1	421.1	1199.5	713.7
Viral load (copies/ml)	501979	922203	151357	169114
Time elapsed since diagnosis (months)	112.2	88.4	102.0	49.4
Treatment time with HAART (yr)	6.0	5.1	6.4	3.4
Treatment time with the last HAART scheme (yr)	4.1	2.5	2.4	0.6

*n*=10/group. Abbrevations: SD, standard deviation; yr, year.

### HAART

A total of eight patients (80%) from each group received the HAART regimen consisting of emtricitabine (FTC), tenofovir (TDF) and efavirenz (EFV). The remaining patients received zidovudine (AZT), lamivudine (3TC) and one protease inhibitor (PI). The average duration of the treatment in the resistant and non-resistant patients was 4.8 and 4.1 years, respectively.

Prior to receiving treatment with FTC/TDF/EFV that is evaluated in the present study, six patients from each group received a combination of two analogous RT inhibitors, most commonly AZT and 3TC, and a PI, and the other four patients (each group) had no access to treatment. No differences in treatment regimens between both groups were observed (Supplementary Material S1).

### miR-15b-5p, miR-16-5p, miR-20a-5p, miR-26a-5p and miR-126-3p are up-regulated in patients without resistance to HAART

Of the 84 miRNAs evaluated (Supplementary Material S2), miR-106b-5p, miR-17-5p, miR-18a-5p and miR-148-3p were selected to normalize the data according to the normalization criteria. The plasma expression levels of miR-15b-5p, miR-16-5p, miR-20a-5p, miR-26a-5p and miR-126-3p were significantly up-regulated in patients without resistance to HAART compared with patients with resistance (*P*<0.05) (Table 2).

**Table 2:** Expression of miRNAs in plasma by PCR array

ID miRNA mature	Fold change	*P*-value	Citations linking these miRNAs in HIV infection
hsa-miR-15b-5p	0.216	*0.03	Reynoso et al. [12]
hsa-miR-16-5p	0.362	*0.05	Munshi et al. [11];
			Reynoso et al. [12]
hsa-miR-19b-3p	0.175	NS	Reynoso et al. [12]
hsa-miR-20a-5p	0.307	*0.04	Reynoso et al. [12]
hsa-miR-21-5p	0.181	NS	Reynoso et al. [12]
hsa-miR-23a-3p	0.134	NS	Reynoso et al. [12]
hsa-miR-26a-5p	0.090	*0.05	
hsa-miR-27a-3p	3.202	NS	Reynoso et al. [12]
hsa-miR-29a-3p	0.463	NS	Reynoso et al. [12]
hsa-miR-92a-3p	0.058	NS	Reynoso et al. [12]
hsa-miR-122-5p	0.228	NS	Triboulet et al. [37]
hsa-miR-126-3p	0.181	*0.04	Reynoso et al. [12]
hsa-miR-146a-5p	0.250	NS	Reynoso et al. [12]
hsa-miR-150-5p	0.404	NS	Munshi et al. [11]
hsa-miR-155-5p	2.772	NS	Reynoso et al. [12]
hsa-miR-191-5p	0.168	NS	Munshi et al. [11]
hsa-miR-210-3p	25.264	NS	Reynoso et al. [12]
hsa-miR-223-3p	0.040	NS	Munshi et al. [11]
			Reynoso et al. [12]

Abbreviation: NS, not significant. *Indicates statistically significant differences (*P*<0.05) by Student’s *t* test for each miRNA in HIV-1 patients with resistance and non-resistance to HAART, and the linked citations state the miRNAs in HIV infection.

### Relative expression levels of selected miRNAs

**Figure 2:**
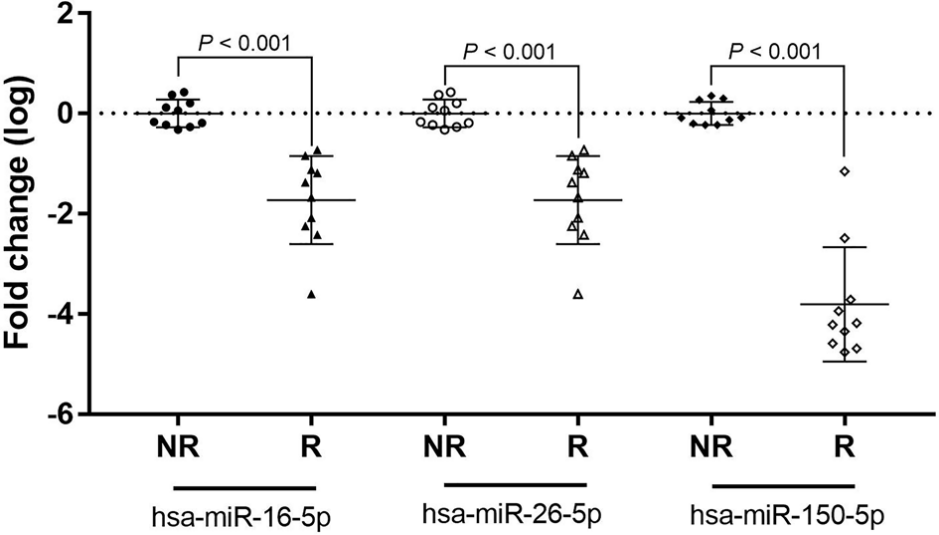
Relative expression fold change (log_10_) of 26a-5p, miR-16-5p and miR-150 Relative expression in plasma from HIV-1 patients with resistance (R) and non-resistance (NR) to HAART. The individual points graph on a log scale, indicate the number of fold change in each miRNA (*P*<0.001).

The three miRNAs selected to repeat the relative quantification in a sample (*n*=10 per group) were miR-16-5p, miR-26a-5p and miR-150. miR-150 has been used as a reference in several HIV studies [11,33,34], while miR-16-5p and miR-26a-5p were selected as they exhibited greater expression than miR-150 in the present study. The detection range of miR-16-5p was higher than that of miR-150; the plasma expression levels of miR-16-5p (0.019 ± 0.021 fold-change), miR-26a-5p (0.001 ± 0.006 fold-change) and miR-150 (0.0002 ± 0.007 fold-change) were down-regulated in patients with resistance to HAART compared with patients without resistance (*P*<0.05; Figure 2).

### Correlations between miRNA expression levels, CD4^+^/CD8^+^ cell counts and viral load

**Figure 3:**
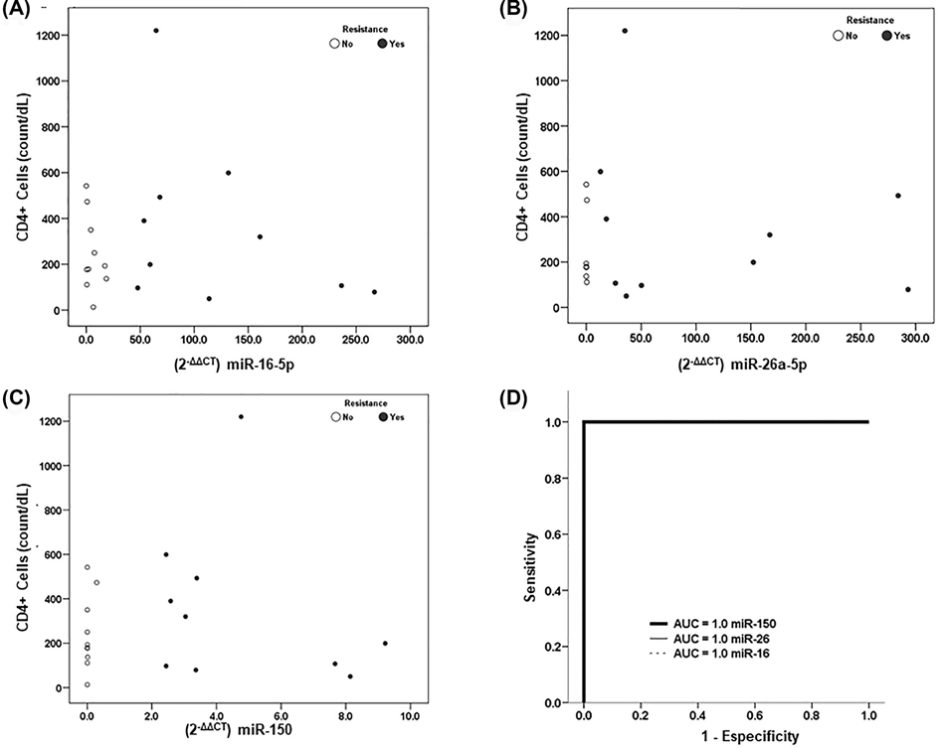
Correlation of miRNA levels (2^−ΔΔ*C*_q_^) and CD4^+^ T cells count (**A**) miR-16-5p, (**B**) miR-26a-5p, (**C**) miR-150 and (**D**) receiver operating characteristic curve analysis of relative expression ratio in plasma of miR-16-5p, miR-26a-5p and miR-150, classified in HIV patients with resistance and non-resistance to HAART.

A correlation between the viral load and fold-change in miR-150 expression level was identified in patients with HAART resistance (r = 0.951; *P*<0.0001). No correlation was found between the levels of miR-16-5p, miR-26a-5p, miR-150 (2^−ΔΔ*C*_q_^) and CD4^+^ count in both groups (Figure 3A–D). However, a correlation was observed between miR-16-5p (2^−ΔΔ*C*_q_^) and CD8^+^ cell count (r = 0.652; *P*<0.003).

The receiver operating characteristic (ROC) curve analysis of the expression ratios of miR-16-5p, miR-26a-5p and miR-150 in patients with or without resistance to HAART revealed an AUC of 1.0 for all three miRNAs evaluated.

### Absolute expression (validation) levels of miRNAs

miR-16-5p was significantly down-regulated in patients with HAART resistance compared with patients without resistance (*P*<0.01; 4.2×10^8^ ± 1.7×10^8^ vs. 9.7×10^9^ ± 2.0×10^9^ copies/ml). Similarly, miR-26a-5p was significantly down-regulated in patients with HAART resistance compared with patients without resistance (*P*<0.05; 2.7×10^6^ ± 1.3×10^6^ vs. 3.7×10^8^±1.2×10^8^ copies/ml). Plasma levels of miR-150 were not quantified due to feasibility issues.

## Discussion

ART is currently the most effective strategy for patients with HIV; however, resistance to one or multiple drugs is common. The HIV-1 viral load is a marker of viral replication and response to ART. However, novel biomarkers to screen for drug resistance are required. HIV-1 replication in CD4^+^ cells and macrophages decreased the expression level of specific miRNAs in HIV-positive patients compared with healthy individuals [15]. Therefore, miRNAs may serve as therapeutic agents or targets in HIV-1 infection.

The present study investigated the expression levels of specific miRNAs and markers of response to ART in HIV-1-positive patients with and without HAART resistance. No differences in patient characteristics, including age, viral load, CD4^+^ and CD8^+^ cell counts, duration of infection and HAART regimen used, were observed between the two groups. However, miRNA expression analysis revealed that specific miRNAs (miR-15b-5p, miR-16-5p, miR-20a-5p, miR-26a -5p and miR-126-3p) were down-regulated in patients with HAART resistance compared with those without resistance, suggesting that miRNAs may be implicated in therapeutic failure.

Liu et al*.* [33] evaluated the expression levels of nine miRNAs in peripheral blood mononuclear cells (PBMCs) in HIV-1-positive patients who did not achieve restoration their expression levels after 6 months of ART (TDF/3TC/EFV); despite the effectiveness of ART in reducing the viral load and increasing the counts of CD4^+^ cells, only miR-150 exhibited a significant correlation with CD4^+^ cell counts among the three miRNAs evaluated (r = 0.6961; *P*=0.0019). However, Liu et al*.* [33] did not investigate miR-16-5p or miR-26a-5p.

A previous study investigated miRNA expression levels in patients with resistance to ART and revealed that miR-150 was the most commonly identified miRNA [11]. However, the array results in the aforementioned study were not different (one of the five plasma samples showed discordant data). Munshi et al*.* [11] demonstrated that miR-150 levels in PBMCs and plasma could distinguish between patients with and without HAART resistance. However, no changes in miR-16-5p levels in PBMCs were observed between patients with and without HAART resistance. In the present study, we found miR-150 exhibited the most significant change in relative expression between patients with and without resistance to ART (*P*<0.05). Nonetheless, the detection range of miR-16-5p was higher than that of miR-150 and was therefore easier to identify.

The diagnostic accuracy was determined by performing ROC analyses. The AUC values of the three markers investigated in the present study were 1.00. The results obtained in the present study were similar to data reported by Munshi et al*.* [11] who revealed high ROC-based AUC values of 0.98 and 0.90 for miRNA-150 expression in PBMCs and plasma (both *P*<0.001), respectively, in patients with ART resistance.

In the present study, lack of correlations between miR16-5p, miR26a-5p and miR-150 levels and the CD4^+^ cell count may be due to a number of factors, including ART resistance, long infection times (∼10 years) and different types of immunological damage (high viral loads and low CD4^+^ cells counts). Reduced expression levels of miR-16-5p and miR-15b-5p have been identified in decreased or restrictive HIV-1 replication in monocytes through an indirect pathway that modulates the viral transcription of purine-rich element binding protein α [15,35], which is involved in HIV infection and immune system function. This may explain the possible immunological failure and the exacerbation of HIV infection in monocytes [17,36].

Reynoso et al*.* [12] demonstrated that plasma expression levels of miR-16-5p and miR-126-3p were up-regulated in chronic patients and elite HIV-infected patients who maintain viral load <50 copies/ml and do not show clinical signs of disease progression during several years, when they were compared with healthy donors. Findings from the present study revealed that HIV-positive patients with HAART resistance had a high viral load, suggesting that the down-regulation of miR-16-5p and miR-126-3p may be associated with viral replication and ART failure. Conversely, increased expression of the aforementioned miRNAs was associated with a reduction in viral load [12].

The other down-regulated miRNAs identified in the present study as miR-15b-5p, miR-20a-5p and miR-26a-5p warrant further investigation. For example, miR-26a-5p and miR-126 activate T lymphocytes *in vitro* through co-stimulation of CD3 and CD28 [37] and miR-26a-5p reduces the differentiation of naïve T cells into T-helper cells 1 or 2 [38]. Furthermore, a previous study revealed that miR-26a-5p is down-regulated in stimulated CD4^+^ cells [39]. Moreover, miR-126-3p is positively correlated with the subsets of CD4^+^ cells, but negatively correlated with infection time, suggesting that this miRNA may affect the progression of HIV and ART failure [12]. Additionally, the decreased expression level of miR-20a-5p in the present study may allow HIV replication through target as transcriptional co-activators of acetyltransferases such as CREB-binding protein-associated factor (PCAF) [15,35,40,41].

The lower relative expression levels of miR-16-5p, miR-26a-5p and miR-150 in ART-resistant patients compared with patients without resistance suggested that therapy failure may be prevented or modulated by miRNAs. Therefore, these miRNAs may serve as potential biomarkers of HIV-1 infections susceptible to ART. Due to the small sample size of the present study, strict normalization criteria were applied; however, the expression levels of miR-16-5p, miR-26a-5p and miR-150 should be evaluated in larger, prospective studies. Future investigations are required to determine the roles of such miRNAs in ART failure and HIV replication.

## Supplementary Material

Supplementary Material S1-S2

## Data Availability

All data generated or analyzed during the present study are included in this published article or available from the corresponding author on reasonable request.

## Abbreviations

AIDS: acquired immunodeficiency syndrome
ART: antiretroviral therapy
ARV: antiretroviral
AUC: area under the curve
Cq: quantification cycle
HAART: highly active antiretroviral therapy
HIV-1: human immunodeficiency virus type 1
miRNA/miR: microRNA
PBMC: peripheral blood mononuclear cell
qPCR: quantitative PCR
RT-qPCR: reverse transcription-qPCR
