# Supplementary material for: Plasma microRNA expression levels in HIV-1-positive patients receiving antiretroviral therapy
Source: Biosci Rep. 2020 May 14;40(5):BSR20194433. doi: 10.1042/BSR20194433 (PMC7225415; doi:10.1042/BSR20194433)
Supplement: Supplementary Material S1-S2 [file BSR-2019-4433_supp.pdf]

Table S1 Comparison of miRNA expression by HAART regimens

Estadísticas de grupo

	Treat	N	Media	Desv. Desviación	Desv. Error promedio
miR-16-5p	FTC+TDF+EFV	16	.6214	.87630	.21907
	AZT+3TC+PI	4	.6558	.47885	.23943
miR-26a-5p	FTC+TDF+EFV	16	.9913	1.66087	.41522
	AZT+3TC+PI	4	.3957	.36128	.18064
miR-150-5p	FTC+TDF+EFV	16	.5699	.79037	.19759
	AZT+3TC+PI	4	.5837	.46852	.23426

FTC+TDF+EFV, (n = 8 / group) versus AZT+3TC+PI (n = 2 / group)

		Prueba de Levene de igualdad de varianzas		prueba t para la igualdad de medias						
									95% de intervalo de confianza de la diferencia	
		F	Sig.	t	gl	Sig. (bilateral)	Diferencia de medias	Diferencia de error estándar	Inferior	Superior
miR-16-5p	Se asumen varianzas iguales	1.429	.247	-.075	18	.941	-.03436	.46034	-1.00151	.93279
	No se asumen varianzas iguales			-.106	8.881	.918	-.03436	.32453	-.77000	.70127
miR-26a-5p	Se asumen varianzas iguales	4.263	.054	.699	18	.493	.59563	.85156	-1.19344	2.38469
	No se asumen varianzas iguales			1.315	17.993	.205	.59563	.45281	-.35572	1.54697
miR-150-5p	Se asumen varianzas iguales	2.046	.170	-.033	18	.974	-.01380	.41727	-.89045	.86285
	No se asumen varianzas iguales			-.045	7.979	.965	-.01380	.30647	-.72083	.69323

There was no statistically significant difference in comparing miRNA expression between groups received the HAART. Regimens consisting of emtricitabine (FTC), tenofovir (TDF) and efavirenz (EFV) *versus* zidovudine (AZT), lamivudine (3TC) and protease inhibitors (PI).

Table S2 General data for patients with non-resistance and resistance to HAART

		Average Cq		Standard Deviation	
Position	Mature ID	Resistence group	Non-resistance group	Resistence group	Non-resistance group
A01	hsa-let-7a-5p	20.422	19.778	2.77155552	6.670447511
A02	hsa-miR-1-3p	23.112	22.038	2.519805548	2.067067972
A03	hsa-miR-100-5p	21.832	20.358	5.389612231	2.620614432
A04	hsa-miR-106b-5p	16.562	15.752	0.887085114	0.816008578
A05	hsa-miR-10b-5p	19.958	22.256	5.663247302	2.292843213
A06	hsa-miR-122-5p	19.776	16.992	2.649052661	3.912258938
A07	hsa-miR-124-3p	20.316	22.102	4.495362054	3.677134754
A08	hsa-miR-125b-5p	21.21	19.35	1.118995085	2.125170581
A09	hsa-miR-126-3p	15.718	12.6	3.69931345	1.476228302
A10	hsa-miR-133a-3p	24.628	26.312	2.408582571	2.415299153
A11	hsa-miR-133b	25.292	24.756	1.894959102	3.463182063
A12	hsa-miR-134-5p	21.036	20.72	0.891924885	1.836926781
B01	hsa-miR-141-3p	23.2	25.188	2.325704194	1.080425842
B02	hsa-miR-143-3p	20.216	20.39	0.548023722	0.721907196
B03	hsa-miR-146a-5p	17.408	14.756	2.273668841	1.900705132
B04	hsa-miR-150-5p	16.156	16.242	5.772701274	2.666621458
B05	hsa-miR-155-5p	20.908	21.728	4.736160893	2.188485778
B06	hsa-miR-17-5p	15.32	15.342	1.382624316	0.804748408
B07	hsa-miR-17-3p	22.14	21.898	1.240866633	0.733668863
B08	hsa-miR-18a-5p	17.834	16.826	0.731354907	0.855178344
B09	hsa-miR-192-5p	18.114	18.002	3.614730972	2.261895223
B10	hsa-miR-195-5p	16.95	12.99	6.231612953	1.074755786
B11	hsa-miR-196a-5p	22.26	24.818	6.17959141	3.118151696
B12	hsa-miR-19a-3p	16.79	15.262	1.329793217	1.080981036
C01	hsa-miR-19b-3p	18.382	15.216	5.432579498	1.144303281
C02	hsa-miR-200a-3p	25.39	25.56	3.180338032	1.48593405
C03	hsa-miR-200b-3p	22.98	22.128	0.997120855	2.108949976
C04	hsa-miR-200c-3p	21.824	22.392	2.444223803	4.918035177
C05	hsa-miR-203a-3p	24.426	25.18	3.117904745	2.878541297
C06	hsa-miR-205-5p	23.298	25.056	3.619097954	1.533649243
C07	hsa-miR-208a-	24.685	26.488	8.011776748	4.541725443

	3p				
C08	hsa-miR-20a-5p	16.388	14.034	2.698206812	1.047201031
C09	hsa-miR-21-5p	15.61	12.492	3.648999863	1.560022436
C10	hsa-miR-210-3p	17.534	21.542	3.437008874	4.356244943
C11	hsa-miR-214-3p	23.51	25.038	3.058463667	1.411407099
C12	hsa-miR-215-5p	24.07	23.618	4.119447779	2.384810265
D01	hsa-miR-221-3p	15.654	14.412	2.119877355	1.6748791
D02	hsa-miR-222-3p	18.468	15.3	5.35422917	1.652392205
D03	hsa-miR-223-3p	16.172	10.858	6.646071772	2.635340585
D04	hsa-miR-224-5p	17.95	18.65	3.638729174	2.733175808
		**Average Cq**		**Standard Deviation**	
**Position**	**Mature ID**	**Resistence group**	**Non-resistance group**	**Resistence group**	**Non-resistance group**
D05	hsa-miR-23a-3p	15.802	12.254	3.641479095	2.136593082
D06	hsa-miR-25-3p	16.712	14.084	1.33460481	2.125365851
D07	hsa-miR-27a-3p	13.236	14.264	3.524248856	0.815125757
D08	hsa-miR-296-5p	21.806	23.83	2.778296241	2.250455509
D09	hsa-miR-29a-3p	18.932	17.17	3.131927522	0.631585307
D10	hsa-miR-30d-5p	16.3	14.34	2.204846933	1.780828459
D11	hsa-miR-34a-5p	21.146	21.282	1.422736799	2.042540085
D12	hsa-miR-375	20.836	21.72	3.859472762	2.06329833
E01	hsa-miR-423-5p	18.578	15.826	5.295027856	2.319219696
E02	hsa-miR-499a-5p	25.348	26.562	8.325005706	1.694526483
E03	hsa-miR-574-3p	20.65	18.838	0.678454125	2.152282509
E04	hsa-miR-885-5p	24.528	22.352	1.130628144	3.039690774
E05	hsa-miR-9-5p	24.168	25.298	3.956787839	1.627181612
E06	hsa-miR-92a-3p	16.578	11.81	5.514695821	2.17637313
E07	hsa-miR-93-5p	16.388	15.1	1.458139225	0.897217922
E08	hsa-let-7c-5p	19.6	18.386	0.596112406	2.453788907
E09	hsa-miR-107	19.632	19.412	2.007802281	1.665674038
E10	hsa-miR-10a-5p	21.004	21.996	3.49086379	2.477302969
E11	hsa-miR-128-3p	18.056	18.846	1.996504445	2.909455275
E12	hsa-miR-130b-3p	19.464	19.632	1.316123095	1.788706236
F01	hsa-miR-145-5p	18.658	18.154	1.679559466	0.653972476
F02	hsa-miR-148a-3p	17.762	16.954	0.77972431	1.417702367
F03	hsa-miR-15a-5p	18.052	18.06	1.007109726	0.393573373
F04	hsa-miR-184	26.126	28.266	3.618263396	3.949237901
F05	hsa-miR-193a-5p	21.448	22.38	1.38396893	3.237491313
F06	hsa-miR-204-5p	22.954	23.618	3.272228598	2.447227002
F07	hsa-miR-206	23.62	25.548	6.572035453	2.32229843
F08	hsa-miR-211-5p	22.532	25.652	11.1951226	2.018853635
F09	hsa-miR-26b-5p	16.688	15.412	0.898871515	1.988283179
F10	hsa-miR-30e-5p	17.148	15.91	2.275844898	0.710703876

F11	hsa-miR-372-3p	27.598	27.984	1.609975155	1.062958137
F12	hsa-miR-373-3p	26.986	26.268	1.624863071	2.899477539
G01	hsa-miR-374a-5p	19.48	17.734	1.118928058	2.519430491
G02	hsa-miR-376c-3p	20.7	20.416	0.983564944	1.360599133
G03	hsa-miR-7-5p	21.56	19.546	2.47655002	2.224922471
G04	hsa-miR-96-5p	20.812	26.968	8.02200536	1.759849425
G05	hsa-miR-103a-3p	16.8	15.696	3.199023288	1.05573671
G06	hsa-miR-15b-5p	17.122	14.262	3.049216293	1.447798328
G07	hsa-miR-16-5p	13.836	11.72	1.559753186	1.069462482
G08	hsa-miR-191-5p	16.28	13.058	4.54014317	1.779991573
G09	hsa-miR-22-3p	14.35	15.51	1.686475615	0.620040321
G10	hsa-miR-24-3p	15.912	13.774	3.043167757	1.52878383
G11	hsa-miR-26a-5p	16.27	12.14	5.109500954	1.557032434

Position	Mature ID	Average Cq		Standard Deviation	
		Resistence group	Non-resistance group	Resistence group	Non-resistance group
G12	hsa-miR-31-5p	25.26	26.996	5.277120427	2.388855374
H02	cel-miR-39-3p	16.268	14.522	2.388497436	0.751212353
H03	SNORD61	20.478	24.462	3.834458241	1.860610115
H04	SNORD68	21.988	23.382	3.275846455	2.538802867
H05	SNORD72	27.726	28.324	2.177826899	1.049895233
H06	SNORD95	20.414	20.77	1.9917781	2.548067895
H07	SNORD96A	22.356	23.168	1.702932764	2.336615073
H08	RNU6-6P	23.938	24.96	0.638725293	1.624115144
H09	miRTC	14.848	14.384	1.699211582	0.937245966
H10	miRTC	14.888	14.494	1.707240463	0.847012397
H11	PPC	14.142	14.68	0.289948271	0.5703946
H12	PPC	14.3	14.772	0.371954298	0.332445484

Abbreviations: ID:Identification; Cq: quantification Cycles.
